# Alpha- and Beta-Cyclodextrin Inclusion Complexes with 5-Fluorouracil: Characterization and Cytotoxic Activity Evaluation

**DOI:** 10.3390/molecules21121644

**Published:** 2016-12-01

**Authors:** Cristina Di Donato, Margherita Lavorgna, Roberto Fattorusso, Carla Isernia, Marina Isidori, Gaetano Malgieri, Concetta Piscitelli, Chiara Russo, Luigi Russo, Rosa Iacovino

**Affiliations:** Department of Environmental, Biological and Pharmaceutical Sciences and Technologies, Second University of Naples, via Antonio Vivaldi 43, 81100 Caserta, Italy; cristina.didonato@unina2.it (C.D.D.); margherita.lavorgna@unina2.it (M.L.); roberto.fattorusso@unina2.it (R.F.); carla.isernia@unina2.it (C.I.); marina.isidori@unina2.it (M.I.); gaetano.malgieri@unina2.it (G.M.); concetta.piscitelli@unina2.it (C.P.); chiara.russo@unina2.it (C.R.); luigi.russo2@unina2.it (L.R.)

**Keywords:** cyclodextrin, 5-fluorouracil, inclusion complex, cytotoxicity

## Abstract

Cyclodextrins are natural macrocyclic oligosaccharides able to form inclusion complexes with a wide variety of guests, affecting their physicochemical and pharmaceutical properties. In order to obtain an improvement of the bioavailability and solubility of 5-fluorouracil, a pyrimidine analogue used as chemotherapeutic agent in the treatment of the colon, liver, and stomac cancers, the drug was complexed with alpha- and beta-cyclodextrin. The inclusion complexes were prepared in the solid state by kneading method and characterized by Fourier transform-infrared (FT-IR) spectroscopy and X-ray powder diffractometry. In solution, the 1:1 stoichiometry for all the inclusion complexes was established by the Job plot method and the binding constants were determined at different pHs by UV-VIS titration. Furthermore, the cytotoxic activity of 5-fluorouracil and its complexation products were evaluated using the 3-(4,5-dimethylthiazol-2-yl)-2,5-diphenyltetrazolium bromide (MTT) assay on MCF-7 (breast cancer cell line), Hep G2 (hepatocyte carcinoma cell line), Caco-2 (colon adenocarcinoma cell line), and A-549 (alveolar basal epithelial carcinoma cell line). The results showed that both inclusion complexes increased the 5-fluorouracil capability of inhibiting cell growth. In particular, 5-fluorouracil complexed with beta-cyclodextrin had the highest cytotoxic activity on MCF-7; with alpha-cyclodextrin the highest cytotoxic activity was observed on A-549. The IC_50_ values were equal to 31 and 73 µM at 72 h, respectively. Our results underline the possibility of using these inclusion complexes in pharmaceutical formulations for improving 5-fluorouracil therapeutic efficacy.

## 1. Introduction

**Figure 1:**
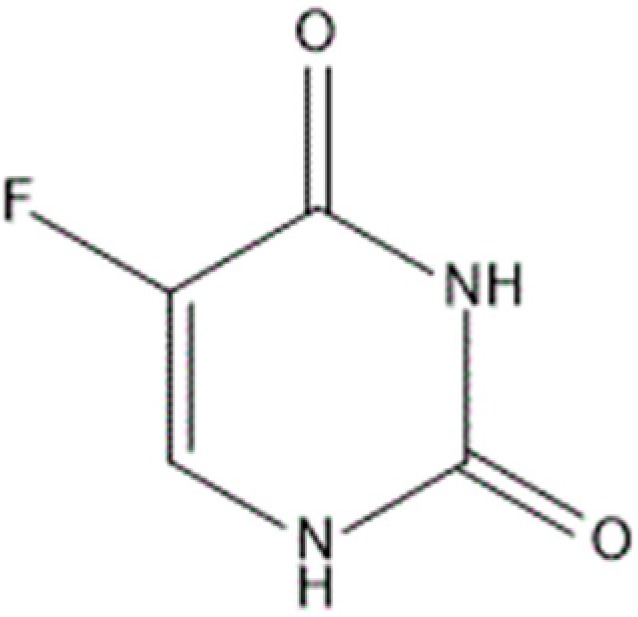
Chemical structure of 5-fluorouracil.

**Figure 2:**
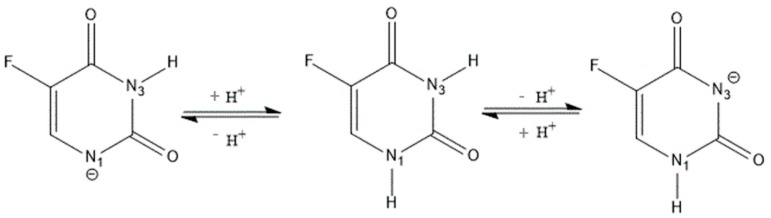
The N1 and N3 deprotonation sites of 5-fluorouracil.

5-fluorouracil (5-FU), 5-fluoro-2,4-(1*H*,3*H*)-pyrimidindione (Figure 1) [1], is a substituted uracil presenting a fluorine atom in the 5-position and its empirical formula is C_4_H_3_FN_2_O_2_. The molecule can be deprotonated in the N1 and N3 positions differently, causing simple correlations between inductive properties and p*K*_a_ values (Figure 2) [2].

5-FU is one of the most widely used agents in cancer therapy [3], producing a good response in colon, rectal, breast, gastrointestinal tract, pancreas, head, and ovarian cancers [4,5,6]. This drug inhibits the thymidylate synthase enzyme resulting in a depletion of thymidine, thus interfering with the incorporation of its metabolites in RNA and DNA [7]. In this way, it is able to shut down the tumor cell growth, arresting malignant cells in the S-phase, and driving them to apoptosis [8]. The cytotoxic activity of 5-FU is time-dependent, according to the short half-life of the drug. This behavior makes it necessary to administer increasing and gradually frequent doses to ensure the achievement of optimal plasma concentrations [9]. Nevertheless, the systemic exposition at high doses of 5-FU can induce serious side effects, such as leukoencephalopathy, stroke [10], diarrhea, and gastrointestinal mucositis [11]. Due to its erratic bioavailability, the most suitable method of administration for 5-FU is in the form of injections or infusions [8], however, the limited aqueous solubility, which is equal to 12.5 mg/mL [12,13], further restricts its use. Therefore, the development of a new strategy for the administration of 5-FU, that will improve its solubility and therapeutic efficacy and, also, decrease the drug allocation in the systemic circulation, is highly desirable. In this context, there are many possible approaches in drug formulation, such as the addition of surfactants and co-solvents, modification of the drug form, and the use of cyclodextrins [14,15]. 

Cyclodextrins (CDs) are cyclic oligosaccharides constituted by six or more glucopyranose units, linked by α-(1,4) bonds and represent versatile complexing agents. They are characterized by a toroidal shape structure, with a hydrophilic external surface and a hydrophobic inner surface, and are able to interact with various organic molecules, forming more soluble and stable host-guest systems with non-covalent bonds [16,17]. Here, we prepared and characterized two inclusion complexes of 5-FU with α-CD (5-FU:αCD) and with β-CD (5-FU:βCD) in order to evaluate how the formation of these inclusion complexes can influence 5-FU bioactivity.

β-CD can easily accommodate aromatic and heterocyclic structures, the complex production could have small costs and be available on market [18]; however, β-CD is less aqueous soluble with respect to alpha and gamma CDs [19]. 

As in literature many articles report the complexation of 5-FU with β-CD or β-CD derivatives [6,20,21] and the β-CD is hemolytic and as such it cannot be used in pharmaceutical formulations for parenteral administration [22], we also considered the formation of 5-FU:αCD inclusion complex. In fact, α-CD has an inner cavity diameter that appears to be suitable to accommodate the 5-FU molecule [23] and, more important, does not present an apoptotic activity against erythrocytes [24]. We studied the cytotoxicity of the obtained inclusion complexes on several cancer cell lines and compared it to the pure compound. 

## 2. Results

### 2.1. Characterization of Inclusion Complexes in Solution

**Figure 3:**
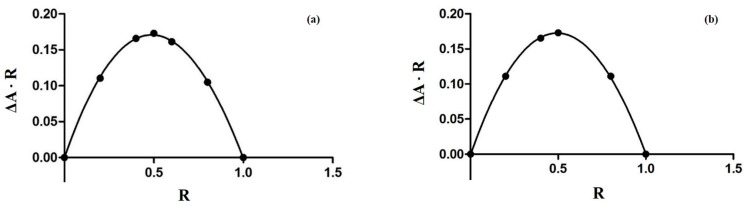
Job plot for the complex 5-FU:αCD (**a**); and for the complex 5-FU:βCD (**b**) at λ = 266 nm.

**Figure 4:**
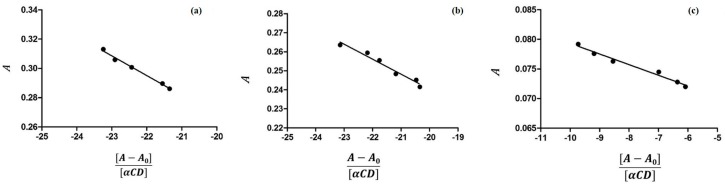
Dependence of 5-FU absorbance from α-CD concentration in aqueous solutions at different pH values: (**a**) pH = 4.3 (λ = 266.6 nm); (**b**) pH = 6.8 (λ = 266.6 nm); and (**c**) pH = 9.8 (λ = 271.2 nm).

**Figure 5:**
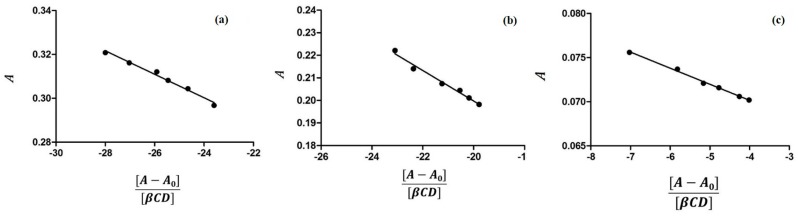
Dependence of 5-FU absorbance from β-CD concentration in aqueous solutions at different pH values: (**a**) pH = 4.3 (λ = 266.2 nm); (**b**) pH = 6.8 (λ = 266.4 nm); and (**c**) pH = 9.8 (λ = 269.8 nm).

The inclusion of the guest molecule inside the cyclodextrinic cavity can deeply change its physicochemical features as the absorbance in the UV-VIS spectrum [25,26]. The 1:1 stoichiometry for both complexes was determined using the Job method [27] considering the maximum of the curve at *R* = 0.5 (Figure 3). Spectroscopic methods were used to evaluate the binding constants (Kbs) to find analytical differences between the free and complexed drug [28]. The 5-FU can be present in solution in two different forms depending on the pH value and each of them may form complexes with CDs. Thus, the inclusion of 5-FU with α-CD and β-CD was studied in buffer solutions at various pHs. In Figure 4 and Figure 5 the results of the dependence of 5-FU absorbance on CD concentrations are shown; the maximum absorption wavelength of 5-FU was pH-dependent. Figure 4 reports the case of α-CD where the maximum is found at 266.6 nm (pH = 4.3), at 266.6 nm (pH = 6.8), and at 271.2 nm (pH = 9.8) respectively. Figure 5 shows the case of β-CD with the maximum at 266.2 nm (pH = 4.3), at 266.4 nm (pH = 6.8) and at 269.8 nm (pH = 9.8). These results suggest that 5-FU:αCD and 5-FU:βCD inclusion complexes were formed. The Kbs for each complex can be obtained from absorbance data using the modified Benesi-Hildebrand Equation (Equation (1) in the Materials and Methods section) [29]. Therefore, a plot of *A* versus (*A* − *A*_0_)/[*H*] gives a straight line with slope equal to −1/Kb. In Table 1 the values of binding constants at different pHs are reported. The results show that the binding constant value is very sensitive to pH [30]; in fact, for the 5-FU:αCD complex we have Kb_9.8_ > Kb_6.8_ > Kb_4.3_, while for 5-FU:βCD complex we have Kb_9.8_ > Kb_4.3_ > Kb_6.8_. These findings suggest that the inclusion of 5-FU molecule inside both, α-CD and β-CD cavities, is more suitable in basic conditions. The basic pH is likely to favor a higher number of hydrogen bonds that stabilize the interaction because of the deprotonated form of the drug. The 5-FU structure has two possible deprotonation sites: N1 and N3 (Figure 2). p*K*_a_ value of the first site corresponds to 9.05, while if the protonation occurs to the second site the p*K*_a_ value is equal to 7.26 [2]. 

### 2.2. Characterization of Inclusion Complexes in Solid State

**Figure 6:**
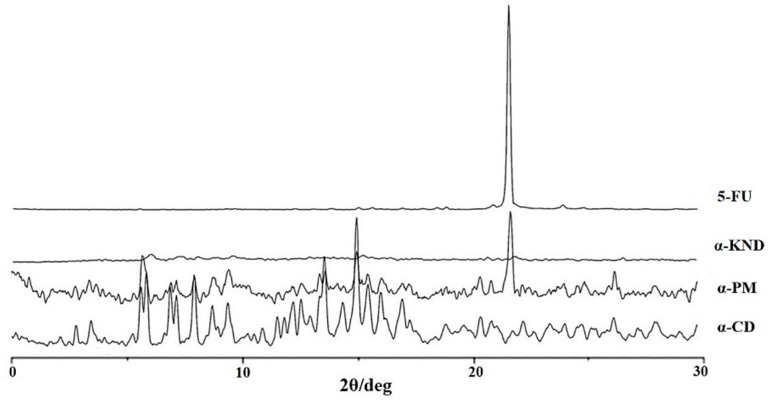
The powder XRD patterns of 5-FU, α-KND, α-PM, and α-CD.

XRD diffraction studies are useful to evidence the formation of inclusion complex comparing crystallinity changes or amorphicity upon host-guest interaction. XRD patterns of pure 5-FU, α-CD, their physical mixture (α-PM), and their kneading product (α-KND) are shown in Figure 6.

**Figure 7:**
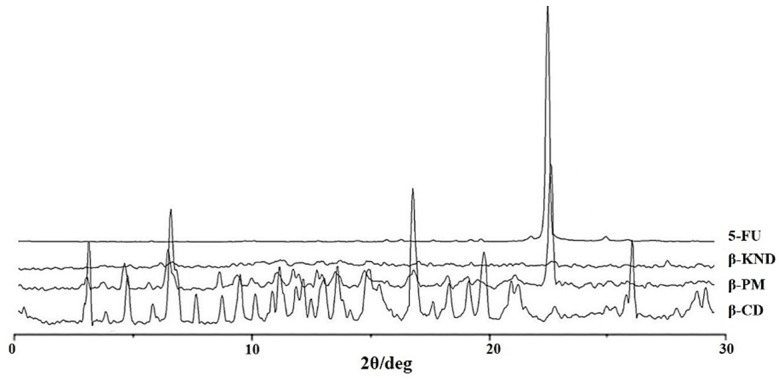
The powder XRD patterns of 5-FU, β-KND, β-PM, and β-CD.

XRD patterns of pure 5-FU, β-CD, their physical mixture (β-PM), and their kneading product (β-KND) are shown in Figure 7.

The XRD patterns of α-CD and β-CD revealed several diffraction peaks, which are indicative of their crystalline character. The characteristic peak of 5-FU appeared at a diffraction angle of 2θ at 28.44°, according to literature data [31]. For the kneading products (α-KND and β-KND) a completely diffuse diffraction pattern was observed, which reveals their amorphous character. Comparing the diffraction patterns of pure components with α-PM and β-PM, it is possible to observe how the characteristic peaks of pure components are still present in the diffractograms of the physical mixtures although with a reduced intensity. 

**Figure 8:**
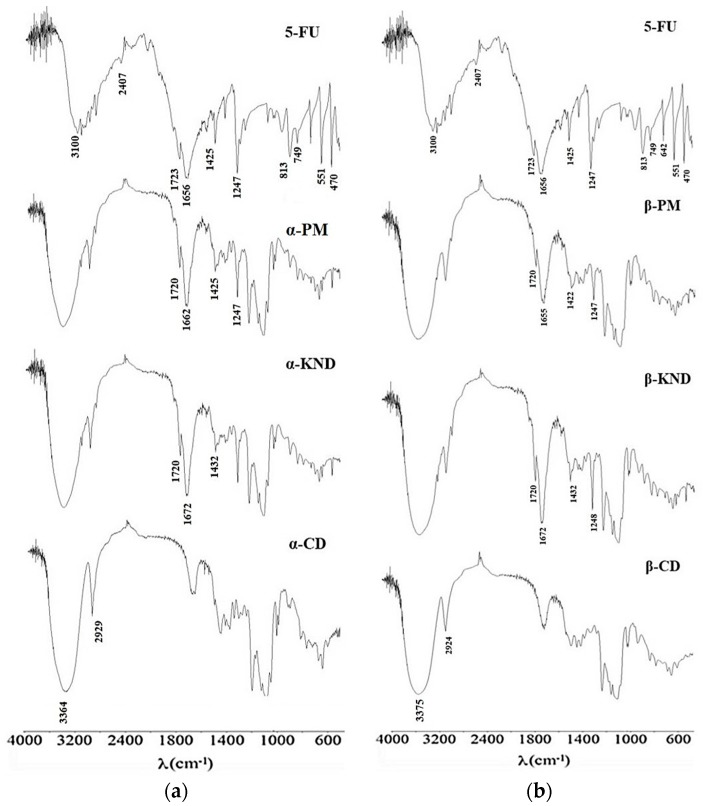
(**a**) FT-IR spectra of 5-FU, α-PM, α-KND, and α-CD; (**b**) FT-IR spectra of 5-FU, β-PM, β-KND, and β-CD.

FT-IR spectroscopy can be used to estimate the interaction between CD and the guest molecules considering that the characteristic bands of the guest can change upon complexation. In Figure 8a are reported the FT-IR spectra of α-CD, 5-FU, α-PM, and α-KND while the Figure 8b shows the spectra of 5-FU, β-CD, β-PM, and β-KND. The spectrum of 5-FU shows characteristic absorption bands in the region between 1656 and 1723 cm^−1^ correlated to the C=C, C=N, C=O, while the region at 1247–1425 cm^−1^ was assigned at the vibration of the substituted pyrimidine. The bands at 470, 551, 642, 749, and 813 cm^−1^, as well as those between 2407 and 3100 cm^−1^ are due to the aromatic ring [6]. The peaks correlated to the aromatic ring for the drug alone are weakened in the spectra of α-PM and β-PM. These peaks show the same weak intensity in the spectra of both KND products; furthermore, the bands in the region between 1656 and 1723 cm^−1^ correlated to the C=C, C=N, and C=O are shifted, while some bands in the region between 2407 and 3100 cm^−1^ correlated to the aromatic ring result disappeared. These changes suggest the formation of the inclusion complexes. 

### 2.3. Molecular Docking Studies

**Figure 9:**
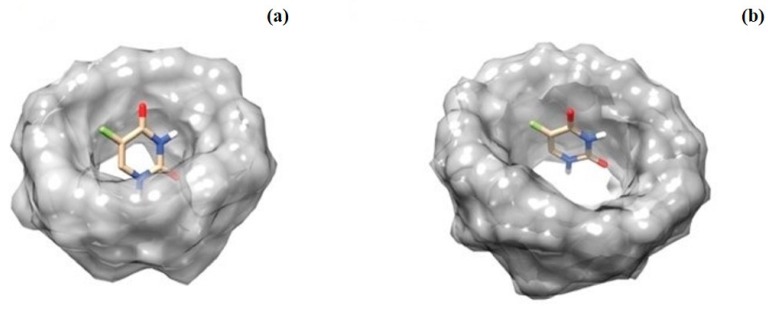
Molecular docking models of the 5-FU in complex with the α-CD (**a**); and β-CD (**b**).

In order to characterize the structural proprieties of the inclusion complexes of the α-CD and β-CD with 5-FU we performed a series of molecular docking (MD) studies. Generally, MD represents a powerful tool to evaluate the binding mode and affinity of an inclusion complex formed by two or more constituent compounds with known structures. The inclusion complexes of α-CD and β-CD with the guest were generated by using Hex software version 6.3 (Team Orpailleur, INRIA Nancy Grand Est, LORIA, 54506 Vavdoeuvre-les-Nancy, France) [32] which is an interactive molecular graphics program for calculating and displaying feasible docking modes of molecules. In particular, the molecular docking protocol involved several steps: the 3D structure of both cyclodexrins and of the 5-FU compound in the protein data bank (PDB) format were downloaded and protonated using the software Reduce [33]; the 3D-optimized structures were stored as PDB files and were used as input in the docking studies; the docking simulations were run, using the parameters described in the Material and Methods section. The docking simulations, in agreement with the UV results indicated that the 5-FU molecule is able to form a 1:1 complex with both cyclodexrins. The selected and optimized molecular docking model obtained for the 5-FU in complex with the α- and β-cyclodextrin is reported in Figure 9. In both cases, the 5-FU compound, entering from the wider rim of the cyclodexrin, is deeply embedded in the hydrophobic cavity. 

### 2.4. Cytotoxicity

**Figure 10:**
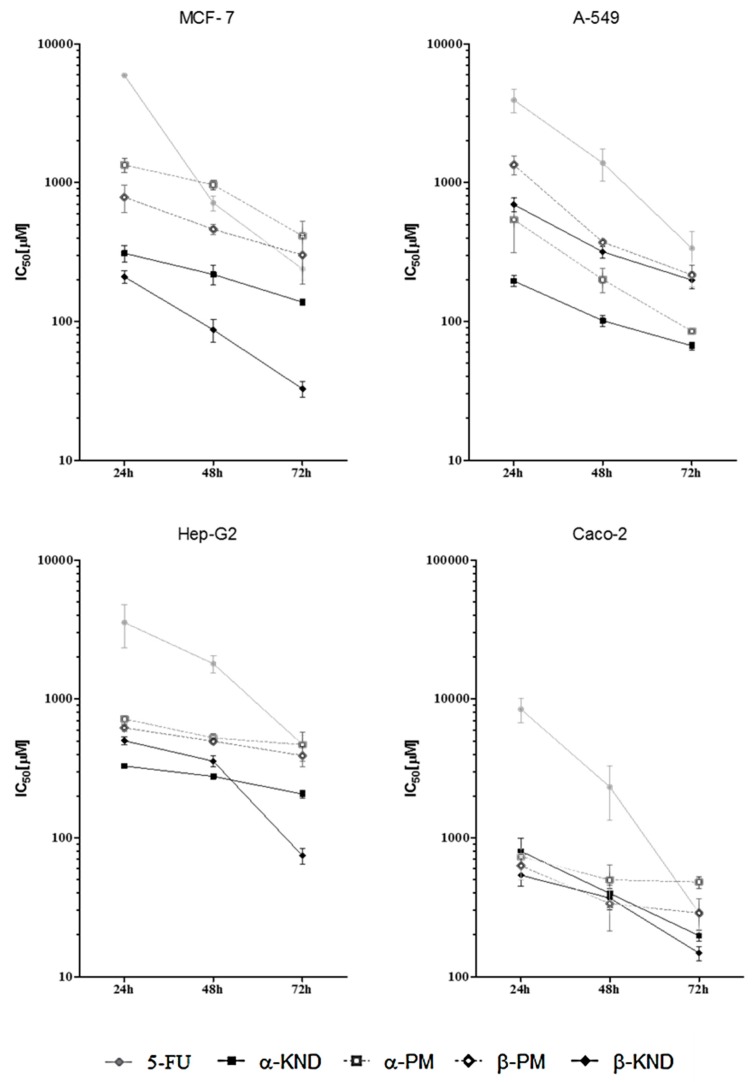
Cytotoxicity expressed as statistically estimated IC_50_ values (μM) of 5-FU, α-KND, β-KND, α-PM, and β-PM on the four selected cell lines at 24, 48, and 72 h. Bars represent standard error.

The cell growth inhibition results of the pure 5-FU, the inclusion complexes α-KND and β-KND, and the physical mixtures α-PM and β-PM against four human cancer cell lines with different estimated IC_50_ values are reported in Table 2. Cytotoxicity, measured as the inhibition of cellular lines viability, was evaluated after 24, 48, and 72 h of incubation (Figure 10 and Table 2).

**Figure 11:**
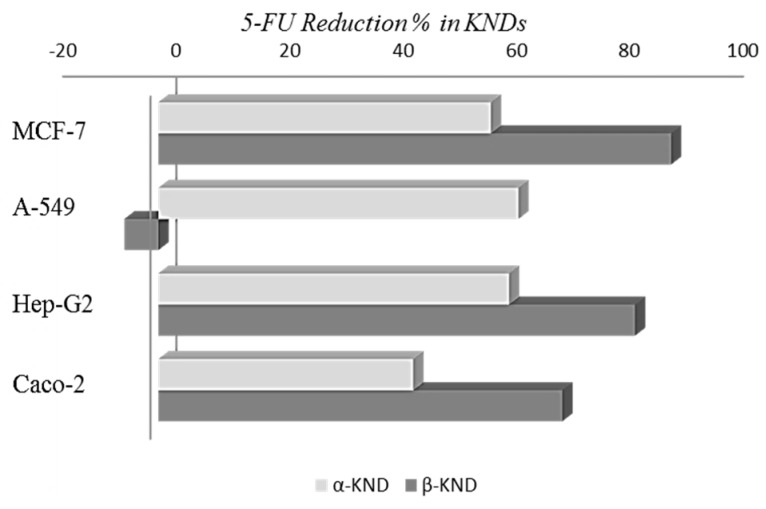
5-FU reduction percentage in KNDs in MCF-7, A549, Hep-G2, and Caco-2 cells at 72 h of exposure to induce the 50% of cellular growth inhibition.

Cell viability of the control was verified using trypan blue and more than 95% of cells were viable. 5-FU alone showed the highest IC_50_ values compared to those showed when this drug was complexed with both cyclodextrins at a molar ratio of CD:5-FU (1:1), indicating substantially higher cytotoxicity of the complexes. The highest cytotoxic effects were found on A-549 for α-KND and on MCF-7 for β-KND. In fact, after 72 h exposition, for the alveolar basal epithelial carcinoma cells, it was necessary to use a concentration of 5-FU equal to 200 µM to obtain the IC_50_ value, while the same effect was reached using only 73 µM of α-KND, with a reduction in the percentage of 5-FU concentration equal to 63.5% (Figure 11).

**Figure 12:**
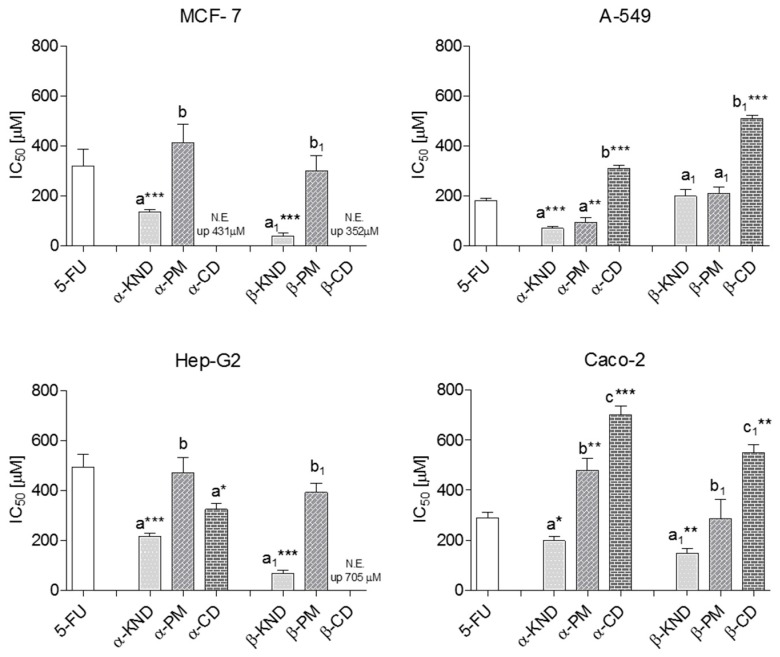
Cytotoxic activity expressed as IC_50_ (μM) of 5-FU, α-KND and β-KND, α-PM and β-PM, α-CD and β-CD against MCF-, A-549, Hep-G2, and Caco-2 cell lines. Significant differences from 5-FU highlighted by asterisks (Dunnett’s test—* *p* < 0.05; ** *p* < 0.01; *** *p* < 0.0001). Different letters mean significant differences for *p* < 0.05 among samples tested (Tukey’s HSD multiple comparison test).

Moreover, for the breast cancer cells it was possible to observe a reduction percentage equal to 90.4% when 5-FU was included in β-CD. After 72 h there was no statistical difference in the cytotoxicity between 5-FU, α-PM, and β-PM, except for α-PM which was more active in A-549 (*p* < 0.01) and less active in Caco-2 (*p* < 0.01), as reported in Figure 12.

In the absence of 5-FU, α-, and β-CD did not show any cytotoxicity on MCF-7 cells at concentrations up to 431 and 352 µM, respectively. The same result was also found for β-CD on Hep-G2 cells up to 705 µM. β-CD showed a certain ability to inhibit the cell growth of the lines A-549 and Caco-2 although it was found at high concentrations. In our experiments, α-CD decreased the viability of Caco-2 showing IC_50_ values equal to 698 µM at 72 h. This result is supported by the study of Ono et al. [35], although these authors showed a decrease in viability at higher concentrations but only after two hours of exposure. The cell lines A-549 and Hep-G2 suffered the co-incubation with α-CD in the MTT assay showing both a growth inhibition at concentrations of 322 and 382 µM, respectively, at 72 h. Our results indicate that the cytotoxicity of the free cyclodextrins differs among cellular lines probably for the differences in the composition of the cell membranes as also suggested by other studies [35,36]. In light of the foregoing, the data showed that the complexation of 5-FU with α-CD and β-CD is able to determine even a dramatically 10-fold increase in the anticancer drug activity, probably ascribable to the enhancement of cellular membranes permeability due to the interaction between CDs and cholesterol contained in cell membranes. In particular, a possible cholesterol extraction by cyclodextrins from lipid rafts would allow an endocytosis mechanism or creation of transient channels leading to the internalization of complexes into cells [37]. Clearly, the different cytotoxic effects of KNDs in different tumoral cells could be due to the different interactions between α-CD and/or β-CD and various cholesterol content in the membranes [35]. On the other hand, as reported by the same authors, the effects of cyclodextrins on the cells could be due to phospholipids that play a key role in the structural properties of membranal layers as carrier of chemicals. Furthermore, as suggested by Gidwani and Vyas, during therapeutic administration, cyclodextrins although potentially harmful at higher doses, extend the residence time of the drugs in site while they quickly disappear from systemic circulation reaching the renal system to be excreted [38]. In this way, they do not exert any effect on drug pharmacokinetics.

## 3. Materials and Methods

All reagents and solvents were of analytical grade and used without prior purification. Double-distilled and MilliQ waters were used throughout the experiments. α-CD was purchased from Alfa Aesar, β-CD was purchased from Sigma-Aldrich. 5-FU (CAS: 51-21-8), 3-(4,5-dimethylthiazol-2-yl)-2,5-diphenyltetrazolium bromide (MTT, CAS: 298-93-1), and 2-propanol (CAS: 67-63-0) were supplied by Sigma-Aldrich (Milan, Italy). Roswell Park Memorial Institute medium (RPMI 1640), Dulbecco’s modified Eagle’s medium phenol red-free (DMEM), fetal bovine serum (FBS), HEPES, l-glutamine, penicillin/streptomycin (10,000 U/mL), and non-essential amino acids (NEAA, 100X) were supplied by Lonza BioWhittaker (Verviers, Belgium). The buffer solutions at different pHs were prepared by adding the appropriate amounts of sodium acetate-acetic acid (pH = 4.3), sodium carbonate-sodium bicarbonate (pH = 9.8), and potassium dihydrogen phosphate (pH = 6.8) according to given procedures [39]; the solutions were prepared just before taking each measurement. The pH values of the buffers were measured using a calibrated CRISON pH-meter Basic 20. MCF-7 and Hep-G2 cell lines were generously offered by Prof. Ciro Abbondanza, the A-549 cell line was offered by Dr. Severina Pacifico, and the Caco-2 cell line was offered by Dr. Nicoletta Potenza, researchers of Second University of Naples, Italy. 

### 3.1. Determination of Binding Constants by UV-VIS Spectroscopy

The molar ratio titration method was used to estimate the binding constants for all the complexes under investigation [40]. α-CD or β-CD solutions at varied concentration (from 0–1.85 mM) were added to a buffered solution of 5-FU at constant concentration (0.05 mM). The absorbances of each obtained solution was measured at different pHs. As the evaluation of Kb by direct spectroscopic methods relies on analytical differences between the free and the complexed drug [41], changes in the absorption intensity of 5-FU was monitored as a function of cyclodextrin concentrations. In the case of α-CD the maximum absorption wavelength of 5-FU was found at 266.6 nm (pH = 4.3), at 266.6 nm (pH = 6.8), and at 271.2 nm (pH = 9.8), while for β-CD at 266.2 nm (pH = 4.3), at 266.4 nm (pH = 6.8), and at 269.8 nm (pH = 9.8). All absorption measurements were made against a blank solution treated in the same way. To conveniently calculate the Kb, we rearranged the Benesi-Hildebrand equation [40] into the straight line form [29] shown in the Equation (1).

(1) $$A=-\frac{1}{Kb}\frac{A-A_{0}}{\left[H\right]}+A_{0}+∆\varepsilon \left[G\right]$$

where *A* and *A*_0_ are the absorbance of 5-FU in the presence and absence of cyclodextrins, respectively, Kb is the stability constant, [*H*] and [*G*] are the concentrations of CD and 5-FU, respectively, and Δε is the difference in the molar absorptivities between the free and complexed guest.

### 3.2. The Job Plot Method for the Determination of Stoichiometry

The stoichiometry of the complexes was determined using the continuous variation method or Job method [27,42]. According to this method, 0.1 mM unbuffered solutions of 5-FU and a solution of CD at same concentration were mixed at different molar ratios *R* = (5-FU)/((5-FU) + (CD)) keeping the volume constant. For each complex, the stoichiometric ratio was obtained by plotting (Δ*A* × *R*) against *R* (where Δ*A* is the absorbance difference of the drug without and with CD) and finding the *R* value corresponding to the maximum of the curve obtained. All measurements were recorded in the wavelength range 200–400 nm at room temperature. For all UV-VIS spectroscopy studies, a UV-1700 spectrometer (Shimadzu, Tokyo, Japan) was used with 1 cm quartz cuvette.

### 3.3. Preparation of Solid Binary System

The 5-FU:αCD and 5-FU:βCD solid binary systems were prepared in 1:1 molar ratio by physical mixing and kneading methods. The physical mixing products (α-PM and β-PM) were prepared using powders of α-CD (0.36 g) or β-CD (0.44 g) and 5-FU (0.05 g). The mixtures were blended in a mortar for 5 min, at room temperature. Kneading compound (α-KND and β-KND) production involved the formation of a paste containing cyclodextrin (α-CD 0.36 g or β-CD 0.44 g), drug molecules (5-FU 0.05 g), and a small volume of a water–methanol (50/50, *v*/*v*) solution. The samples were dried at 40 °C in an oven for 30 min to remove traces of solvent and pulverized.

### 3.4. X-ray Powder Diffraction (XRD)

X-ray powder diffraction (XRD) diffraction patterns were obtained at room temperature using a Bruker AXS D8 Advance diffractometer (Karlsruhe, Germany) with tube anode Cu and a graphite monochromator. Analysis was performed at generator voltage of 40 kV and a current of 30 mA. The diffractograms were recorded in the 2θ angle range between 3° and 30° and process parameters with scanning speed 0.01 θ/s.

### 3.5. Fourier Transform Infrared (FT-IR) Spectroscopy

FT-IR spectra were obtained using KBr disks on a Perkin Elmer Spectrum GX spectrometer (Waltham, MA, USA). The scanning range was kept from 4000 to 400 cm^−1^ with a resolution of 1 cm^−1^.

### 3.6. Molecular Docking Studies

The molecular docking studies of the inclusion complexes of α-CD and β-CD with the guest were performed using the software Hex version 6.3 [32]. The PDB files of α-CD, β-CD and 5-FU were uploaded as inputs into Hex and treated as a receptor (cyclodexrin) and a ligand (5-FU). All input files were analyzed using the spherical harmonic surface of Hex. Computations were performed using the shape complementary scoring function, with 16 and 30 expansion orders for the initial and final steps. The full list of parameters is given in Table 3. In each molecular docking calculation the cyclodexrin was kept as a fixed truncated-cone and the 5-FU was allowed to freely move. Structure refinement and energy minimization were performed with Hex itself. Molecular docking results were clustered into different group based on the root mean-square deviation values at atomic position in the inclusion complex. The lowest energy host-guest inclusion complex conformation was selected and analyzed. Each inclusion complex was analyzed and visualized using MolMol [43], PyMol [44], and CHIMERA [45].

### 3.7. MTT Assay

The cytotoxic activities of CDs, the pure 5-FU, the inclusion complexes obtained by the kneading method (α-KND and β-KND) and physical mixtures (α-PM and β-PM) were tested against the human cancer cell lines using the colorimetric MTT assay, following Baharum et al. [46], with minor modifications [34]. The stock solutions of all samples for cytotoxicity tests were prepared in deionized water at a maximum concentration of 1000 mg/L (Elix 10, Millipore, Milan, Italy). The solutions were tested starting from 800 or 400 mg/L, arranged in a geometric series of five-six concentrations with a dilution factor equal to 2 in DMEM, supplemented with antibiotics and FBS, immediately before tests. Cell lines were maintained in growth medium, consisted of RPMI 1640 with 10% FBS, 2% HEPES, 2% l-glutamine and 1% penicillin/streptomycin. Only for the Caco-2 cell line, 1% NEAA was added to the growth medium. Cells were cultured in tissue culture flasks in a 95% air/5% CO_2_ incubator at 37 °C under saturating humidity. After reaching the 80%–90% of confluence, cells were collected from maintenance cultures and counted with the vital dye, trypan blue, using an optical microscope. Cells (10^4^/well) were seeded in quadruplicate in 100 μL of DMEM/well in 96-well microplates. After 24 h of incubation, the DMEM was removed and 200 μL of fresh medium containing the different concentrations of all samples tested was added to each well and incubated for 24, 48, and 72 h at 37 °C in a CO_2_ incubator. Negative control wells contained DMEM without samples. After 24, 48, and 72 h, 20 μL of yellow MTT solution (5 mg/mL) was added to each well and the cells were incubated for further 4 h at 37 °C. Then, the purple formazan crystals obtained by mitochondrial reduction of MTT were dissolved with 100 μL of 2-propanol. The absorbance was recorded at 590 nm using an Ultra Multifunctional Microplate Reader (TECAN). Cell inhibitory rate was calculated according to the Equation (2):

(2) $$1-\frac{sample\;absorbance}{control\;absorbance}\times 100$$

The results derived from three independent experiments were statistically analyzed by Prism 5 (GraphPad Inc., San Diego, CA, USA). IC_50_ values (concentrations which cause 50% inhibition of cell growth) were calculated by nonlinear concentration/response regression model. ANOVA and Dunnett’s multiple comparisons test estimated the significant differences between 5-FU alone and all other samples. The significant differences among tested samples were determined by Tukey’s multiple comparison test.

## 4. Conclusions

In the present study we have prepared and characterized both in the solid state and in solution the inclusion complexes of a well-known chemotherapeutic agent, the 5-FU, with α-CD and β-CD. The formation of both inclusion complexes was confirmed by Fourier transform-infrared spectroscopy and X-ray powder diffractometry. In aqueous solution, the 1:1 stoichiometry for both the complexes was established via a Job plot and their binding constants evaluated at different pHs. The inclusion complexes prepared were, thus, evaluated in terms of cytotoxic activity using the MTT assay on four different cancer cell lines: MCF-7, A-549, Hep-G2, and Caco-2. Important improvements of the 5-FU cytotoxic effects were found on A-549 upon complexation of the drug with α-CD and on MCF-7 upon complexation with β-CD. These data, according also to the literature data, suggest an enhancement of cellular membranes permeability leading to the internalization of drug into cells. The selective cytotoxic effects of the two kneading products versus different tumoral cells suggest diverse interaction capabilities of α-CD and/or β-CD with the various cholesterol content in the membranes. In order to consider these complexes for future new formulations, β-CD hemoliticity suggests a possible oral administration of its complex with 5-FU while α-CD complexes appear more suitable for parenteral administration.

Overall, we show how the complexation of 5-FU with α-CD or β-CD is able to determine significant increase in the anticancer activity of this widely used drug. Our results complement previous studies on the advantageous reduction of both therapeutic doses and correlated systemic side effects. 

## Figures and Tables

**Table 1:** The binding constants of 5-FU:αCD and 5-FU:βCD evaluated by absorbance measurements at different pH values.

pH	Kb (M^−1^) 5-FU:α-CD	Kb (M^−1^) 5-FU:β-CD
4.3	74	187
6.8	127	148
9.8	563	549

**Table 2:** IC_50_ values of cytotoxicity tests on MCF-7, A-549, Hep-G2, and Caco-2 cell lines for 5-FU, α-KND, β-KND, α-PM, and β-PM in μM with 95% confidence range (in brackets) from 24 to 72 h.

Compound	t (h)	5-FU	α-KND	α-PM	β-KND	β-PM
**MCF-7**	24 h	5 × 10^3^ (3 × 10^3^–8 × 10^3^)	312 (214–455)	>756 ^b^	199 (131–301)	>632 ^b^
	48 h	738 ^a^ (424–1286)	301 (87–400)	>756 ^b^	85 (55–130)	511 (222–1178)
	72 h	324 ^a^ (161–650)	134 (84–216)	463 (217–984)	31 (18–55)	309 (141–676)
**A-549**	24 h	3 × 10^3^ (2 × 10^3^–5 × 10^3^)	207 (116–371)	419 (251–699)	902 (375–1169)	1 × 10^3^ (0.8 × 10^3^–2 × 10^3^)
	48 h	1 × 10^3^ (0.6 × 10^3^–2 × 10^3^)	111 (69–179)	240 (137–423)	334 (220–509)	373 (140–648)
	72 h	200 (153–328)	73 (54–99)	85 (43–170)	212 (108–417)	255 (143–731)
**Hep-G2**	24 h	7 × 10^3^ (2 × 10^3^–24 × 10^3^)	328 (309–346)	732 (678–790)	514 (1531–722)	609 (535–693)
	48 h	2 × 10^3^ (1 × 10^3^–4 × 10^3^)	278 (251–307)	528 (427–654)	414 (178–987)	493 (384–635)
	72 h	590 (380–930)	225 (197–257)	478 (290–786)	94 (58–150)	395 (298–523)
**Caco-2**	24 h	10 × 10^3^ (4 × 10^3^–26 × 10^3^)	700 (480–1010)	920 (460–1810)	600 (390–920)	800 (440–1470)
	48 h	1 × 10^3^ (0.7 × 10^3^–4 × 10^3^)	440 (226–760)	580 (210–1610)	400 (230–700)	350 (140–910)
	72 h	327 (223–480)	180 (140–240)	470 (319–693)	140 (100–190)	327 (224–479)

^a^ Cytotoxicity results of 5-FU on MCF-7 were extracted from Parrella et al. [34]; ^b^ Maximum concentration tested.

**Table 3:** Hex parameters used in this study.

Correlation Type	Shape Only
FFT Mode	3D fast life
Post processing	MM Minimization
Receptor range angle	180
Ligand range angle	180
Twist range	360
Distance range	40
Docking main scan	18
Docking main search	25
